# Development of nomogram models of inflammatory markers based on clinical database to predict prognosis for hepatocellular carcinoma after surgical resection

**DOI:** 10.1186/s12885-022-09345-2

**Published:** 2022-03-07

**Authors:** Shuqi Mao, Xi Yu, Jihan Sun, Yong Yang, Yuying Shan, Jiannan Sun, Joseph Mugaanyi, Rui Fan, Shengdong Wu, Caide Lu

**Affiliations:** 1 Department of Hepatopancreatobiliary Surgery, Ningbo Medical Center Lihuili Hospital, Ningbo University, 315040 Ningbo, Zhejiang China; 2 Medical quality management office, Ningbo Medical Center Lihuili Hospital, Ningbo University, 315040 Ningbo, Zhejiang China

**Keywords:** Hepatocellular carcinoma, Inflammatory marker, Survival, Recurrence, Nomogram

## Abstract

**Background:**

Inflammation plays a significant role in tumour development, progression, and metastasis. In this study, we focused on comparing the predictive potential of inflammatory markers for overall survival (OS), recurrence-free survival (RFS), and 1- and 2-year RFS in hepatocellular carcinoma (HCC) patients.

**Methods:**

A total of 360 HCC patients were included in this study. A LASSO regression analysis model was used for data dimensionality reduction and element selection. Univariate and multivariate Cox regression analyses were performed to identify the independent risk factors for HCC prognosis. Nomogram prediction models were established and decision curve analysis (DCA) was conducted to determine the clinical utility of the nomogram model.

**Results:**

Multivariate Cox regression analysis indicated that the prognostic nutritional index (PNI) and neutrophil-to-lymphocyte ratio (NLR) were independent prognostic factors of OS, and aspartate aminotransferase-to-platelet ratio (APRI) was a common independent prognostic factor among RFS, 1-year RFS, and 2-year RFS. The systemic inflammation response index (SIRI) was an independent prognostic factor for 1-year RFS in HCC patients after curative resection. Nomograms established and achieved a better concordance index of 0.772(95% CI: 0.730-0.814), 0.774(95% CI: 0.734-0.815), 0.809(95% CI: 0.766-0.852), and 0.756(95% CI: 0.696-0.816) in predicting OS, RFS, 1-year RFS, and 2-year RFS respectively. The risk scores calculated by nomogram models divided HCC patients into high-, moderate- and low-risk groups (*P* < 0.05). DCA analysis revealed that the nomogram models could augment net benefits and exhibited a wider range of threshold probabilities in the prediction of HCC prognosis.

**Conclusions:**

The nomograms showed high predictive accuracy for OS, RFS, 1-year RFS, and 2-year RFS in HCC patients after surgical resection. The nomograms could be useful clinical tools to guide a rational and personalized treatment approach and prognosis judgement.

## Background

The Global cancer statistics 2020 report indicated that primary liver cancer is the sixth most commonly diagnosed cancer and the third leading cause of cancer-related death in the world, and 75-85% of primary liver cancer was hepatocellular carcinoma (HCC) [1]. Although HCC can be treated by surgical resection, ablation, and liver transplantation in the early stage, the five-year recurrence rate is as high as 70% and the long-term prognosis is still not ideal [2]. In China, the age-standardized 5-year relative survival of HCC was only 12.1% [3], and only a small proportion of patients with HCC are suited for curative surgery. For operable HCC, recurrence and metastasis were the main drivers of poor prognosis [4], which is the main cause of death in long-term follow-up assessments. The high incidence of intrahepatic recurrence remains a major challenge in HCC therapy, and the prognosis for patients with early recurrence is more aggressive and worse [5].

The majority of HCC cases are associated with chronic inflammation and fibrosis of varying aetiologies, including viral infection hepatitis, alcoholic and nonalcoholic fatty liver disease and toxins. Chronic inflammation induces changes especially in the hepatic immune system, damages hepatic epithelial cells, and allows tumour cells to easily evade immune surveillance [6]. A handful of inflammatory markers easily calculated by preoperative complete blood count and liver function test have been reported to be associated with HCC patient survival post curative surgical resection. The inflammatory marker prognostic nutritional index (PNI) [7], aspartate aminotransferase-to-platelet ratio index (APRI) [8], aspartate aminotransferase-to-lymphocyte ratio index (ALRI) [9], aspartate aminotransferase-to-neutrophil ratio index (ANRI) [10], systemic immune-inflammation index (SII) [11], neutrophil-to-lymphocyte ratio (NLR) [12, 13], platelet to lymphocyte ratio(PLR) [12], monocyte-to-lymphocyte ratio (MLR) [14, 15] and systemic inflammatory response index (SIRI) [16] have been shown to be potential prognostic factors in HCC patients. These inflammatory markers are reflective of the underlying immune health and inflammation status of the patients. However, few studies have constructed clinical nomogram models integrating the aforementioned inflammatory markers to predict the prognosis of HCC.

Accurate prognosis estimation can help surgeons choose appropriate therapeutic measures for HCC patients based on a risk-benefit assessment. Nomogram has been considered a reliable tool to integrate and quantify significant risk factors for disease prognosis [17, 18]. Therefore, we aimed to develop nomogram models of inflammatory markers to predict the risk of overall survival (OS), recurrence-free survival (RFS), 1-year RFS, and 2-year RFS in HCC patients after surgical resection.

## Materials and methods

### Patients

A total of 360 HCC patients who underwent hepatic resection at Ningbo Medical Center Lihuili Hospital from September 2015 to January 2021 were included. The inclusion criteria were as follows: (1) pathologically diagnosed HCC; (2) Child-Pugh A or B classification; (3) no evidence of extrahepatic metastasis; (4) treatment by intended cure resection, which was defined as negative margins with no residual tumour based on the histological examination. The exclusion criteria were as follows: (1) received preoperative anti-cancer medication; (2) history of other cancers; and (3) incomplete clinical or follow-up data. One hundred and sixty-four patients (113 MVI positive and 51 MVI negative) underwent preventive transcatheter arterial chemoembolization (TACE) after hepatectomy as adjuvant therapy, ten patients underwent radiofrequency ablation, one patient underwent chemotherapy, twenty-one patients underwent molecular-targeted drug(sorafenib or lenvatinib) after recurrence. The study was approved by the ethics committee of Ningbo Medical Center Lihuili Hospital (Approval number: KY2021PJ218). All research procedures were in compliance with the relevant guidelines and regulations. Informed consent was obtained from all patients prior to inclusion.

### Laboratory examination and followed-up

Laboratory examinations included blood biochemistry, complete blood count and pathological examination. The albumin-bilirubin (ALBI) score was computed by the formula, -0.085 × (albumin g/l) + 0.66 × log (bilirubin µmol/l) [19]. The inflammatory markers were calculated by the following formula: PNI = serum albumin(g/L) + 5 × lymphocyte(10^9^/L) [20], SII = platelet × neutrophil/lymphocyte [11], NLR = neutrophil/lymphocyte [12], PLR = platelet/lymphocyte [12], MLR = monocyte/lymphocyte [15], SIRI = monocyte × neutrophil/lymphocyte [16], APRI = [aspartate aminotransferase(U/L)/upper limit of normal value (U/L)/platelet] × 100 [9], ANRI = aspartate aminotransferase(U/L)/neutrophil [10], ALRI = aspartate aminotransferase(U/L)/lymphocyte [9].

The cut-off value of biomarkers was set according to the Health Industry Standard of the People’s Republic of China published by National Health Commission of the People’s Republic of China. The cut-off value of AFP was set to 400 ng/mL after reviewing previous HCC studies [21, 22]. Patients were defined as hypertensive based on the ‘gold standard’. Type 2 diabetes mellitus (T2DM) was diagnosed according to the World Health Organization criteria. Anatomic or nonanatomic resection was performed after the clinical evaluation, and all the obtained surgical specimens were histologically assessed by different pathologists.

Baseline data were collected from our clinical database. HCC patients were consistently followed-up after curative resection at intervals of three months. Follow-up was aimed at determination of overall survival (OS) and recurrence-free survival (RFS). All patients were followed up by imaging techniques after treatment to the time of death or last follow-up (US and/or CT scan every 3 months during the first 1 year after surgery and every 3 or 6 months after). Patients also received a routine liver function review, and serum AFP analysis during follow-up visits. OS was measured from the date of curative resection to the date of death or last follow-up visit. RFS was calculated from the date of curative resection to the date when tumour recurrence was diagnosed. The preoperative and tumour recurrence diagnoses were based on criteria set forth in the guidelines for the diagnosis and treatment of primary liver cancer in China [23].

### Statistical analysis

Quantitative variables are reported as medians and interquartile ranges or means and standard deviations, while categorical variables are presented as absolute counts and percentages. Survival curves were calculated using the Kaplan–Meier method and compared with log-rank test. LASSO regression analysis was used for data dimensionality reduction and element selection. Independent prognostic factors of overall survival and tumour recurrence were identified by univariate and multivariate Cox proportional hazards regression. Subsequently, a nomogram was formulated to predict the prognosis of HCC patients. Nomogram performance was assessed via internal validation and calibration curve statistics(concordance index was calculated to measure discrimination with 1000 bootstrapping techniques). Each patient had a total risk score (NomoScore: nomogram risk score) for risk stratification of OS, RFS, 1-year RFS, and 2-year RFS according to nomogram models. Patients were divided into different risk groups (Low-; Moderate-; High-) with the cut-off points automatically calculated using X-tile software (version 3.6.1; Yale University, New Haven, CT, USA) [24]. Decision curve analysis (DCA) was conducted to determine the clinical benefit of the nomogram by quantifying the net benefits along with the increase of threshold probabilities.

Survival analysis, univariate and multivariate Cox regression analyses were performed using SPSS 25.0 (IBM Corporation, 2020, USA). LASSO regression, nomogram, DCA, and survival figures were performed or plotted using R version 3.6.2 (http://www.r-project.org/), with package dependencies: “rms”, “glamet”, “ggDCA”, “rmda” “survival”, “survminer”, and “ggpubr”. *P* < 0.05 was considered statistically significant.

## Results

### Baseline characteristic prognostic outcomes of enrolled patients

In this research, 360 HCC patients who met the inclusion criteria were enrolled from the 518 pathologically diagnosed patients. The median follow-up time was 24 months (1-61 months). The 1-, 3-, and 5-year overall survival rates were 94.7%, 76.2%, and 72.0% respectively. The 1-, 2-, and 3-year recurrence-free rates were 75.3%, 62.7%, and 56.5%, respectively. All baseline characteristics are summarized in Table 1.

**Table 1:** Characteristics of HCC patients

Variables	Median (range)/Mean±SD/N (%)
Gender(male/female)	298(82.8%)/62(17.2%)
Age,years	59.2±10.9
HBV(yes/no)	311(86.4%)/49(13.6%)
Anti-HBV(yes/no)	129(35.8%)/231(64.2%)
Family history of HCC	16(4.4%)/344(95.6%)
Hypertension(yes/no)	101(28.1%)/259(71.9%)
Diabetes(yes/no)	47(13.1%)/313(86.9%)
ALT, U/L (≤50/>50)	287(79.7%)/73(20.3%)
ALP, U/L (≤125/>125)	286(79.4%)/74(20.6%)
GGT, U/L (≤60/>60)	204(56.7%)/156(43.3%)
PT, seconds (≤13/>13)	303(84.2%)/57(15.8%)
ALBI(≤-2.60/ -2.60 to -1.39/>-1.39)	236(65.6%)/120(33.3%)/4(1.1%)
DB, umol/l (≤8/>8)	297(82.5%)/63(17.5%)
INR(≤1/>1)	85(23.6%)/275(76.4%)
AFP,ng/mL (≤20/20 to 400/>400)	167(46.4%)/96(26.7%)/97(26.9%)
PNI	48.9(26.5,73.3)
APRI	0.22(0.04,4.58)
ALRI	21.70(5.67,514.62)
ANRI	10.72(1.51,209.06)
SII	296.59(50.80,7846.67)
NLR	2.00(0.40,73.33)
PLR	102.18(30.91,500.00)
MLR	0.29(0.12,5.17)
SIRI	0.85(0.21,75.43)
Pathological differentiation(well/moderate/poor)	167(46.4%)/96(26.7%)/97(26.9%)
MVI(yes/no)	180(50%)/180(50%)
Cirrhosis(yes/no)	173(48.1%)/187(51.9%)
Number of tumours(solitary/multiple)	290(80.6%)/70(19.4%)
Tumour diameter, cm	4.98±3.07
Tumour capsule(yes/no)	299(83.1%)/61(16.9%)
PVTT(yes/no)	30(8.3%)/330(91.7%)
Child-Pugh grade (A/B)	354(98.3%)/6(1.7%)
AJCC T stage (I - II /III - IV)	280(77.8%)/80(22.2%)

*Abbreviations: HBV* Hepatitis B Virus, *ALT* alanine aminotransferase, *ALP*alkaline phosphatase, *GGT* γ-glutamyl transpeptidase, *PT* prothrombin time, *DB* direct bilirubin, *INR* international normalized ratio, *AFP* alpha-fetoprotein, *MVI* microvascular invasion, *PVTT* portal vein tumour thrombosis

### Dimensionality reduction and element selection

**Fig. 1:**
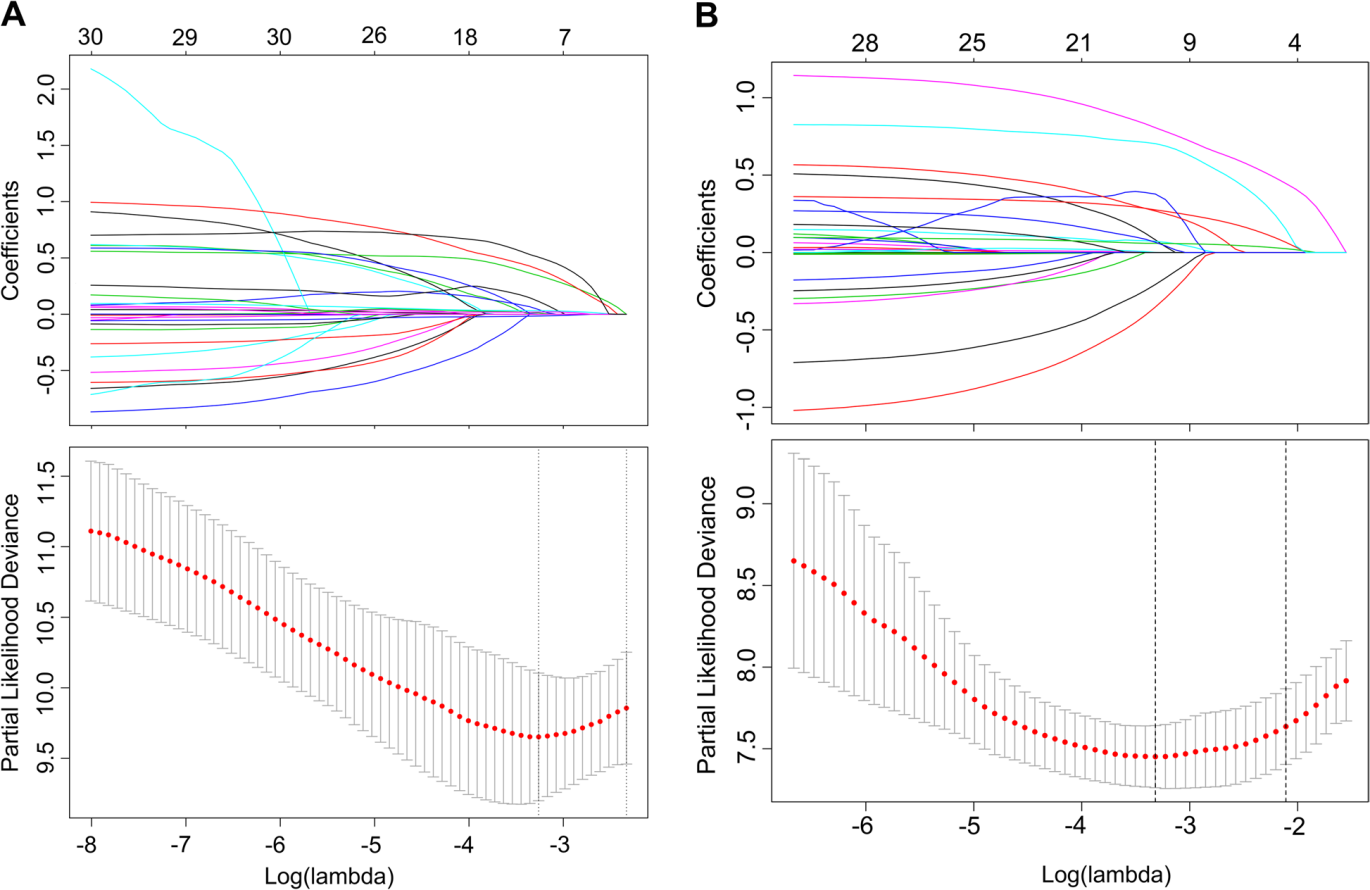
Nomogram model elements selection of OS and RFS using the LASSO regression model. elements selection of OS (**A**) and elements selection of RFS (**B**)

**Fig. 2:**
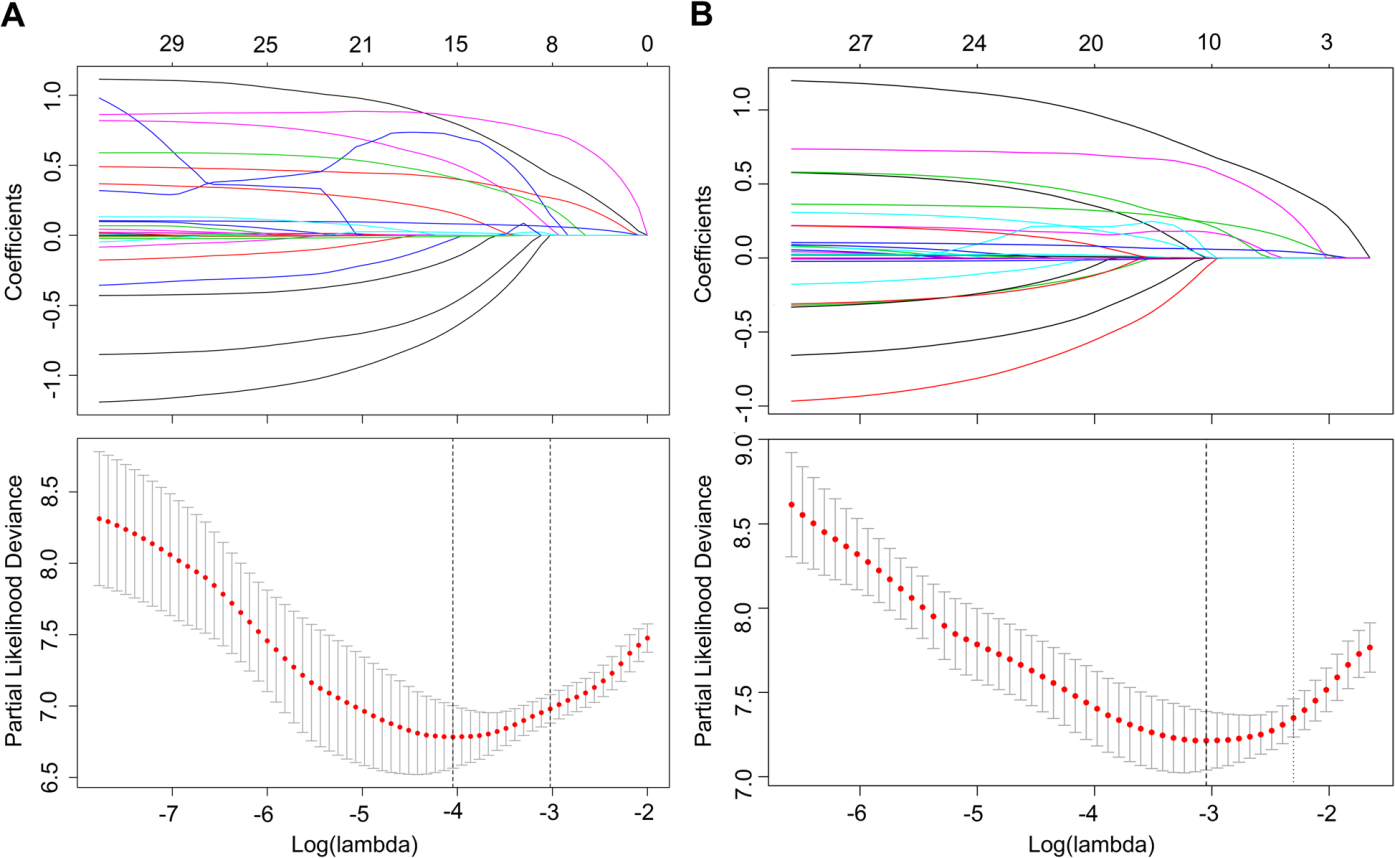
Nomogram model elements selection of 1-year and 2-year RFS using the LASSO regression model. elements selection of 1-year RFS (**A**) and elements selection of 2-year RFS (**B**)

The LASSO coefficient profiles of the features were plotted. The optimum parameter (lambda) selection in the LASSO model performed tenfold cross-validation through minimum criteria. The partial likelihood deviance (binomial deviance) curve is presented versus log (lambda). Dotted vertical lines are shown at the optimum values by performing lambda.min and lambda.1se. Finally, we chose the optimum value corresponding to the minimum value of lambda. In the OS analysis, GGT, DB, AFP, PNI, NLR, SIRI, MVI, tumour diameter, and PVTT were selected using LASSO regression analysis (Fig. 1A). In the RFS analysis, gender, diabetes, ALT, ALBI, AFP, APRI, ANRI, SIRI, MVI, no. of tumour, tumour diameter, tumour capsule, and PVTT were selected (Fig. 1B). In the 2-year RFS analysis, ALT, ALBI, AFP, PNI, APRI, MVI, number of tumour, tumour diameter, tumour capsule, and PVTT were selected (Fig. 2A). In the 1-year RFS analysis, gender, diabetes, ALT, PT, INR, AFP, PNI, APRI, ANRI, SIRI, MVI, cirrhosis, number of tumour, tumour diameter, and PVTT were selected (Fig. 2B).

### Development and validation of the prognosis-predicting nomogram

**Fig. 3:**
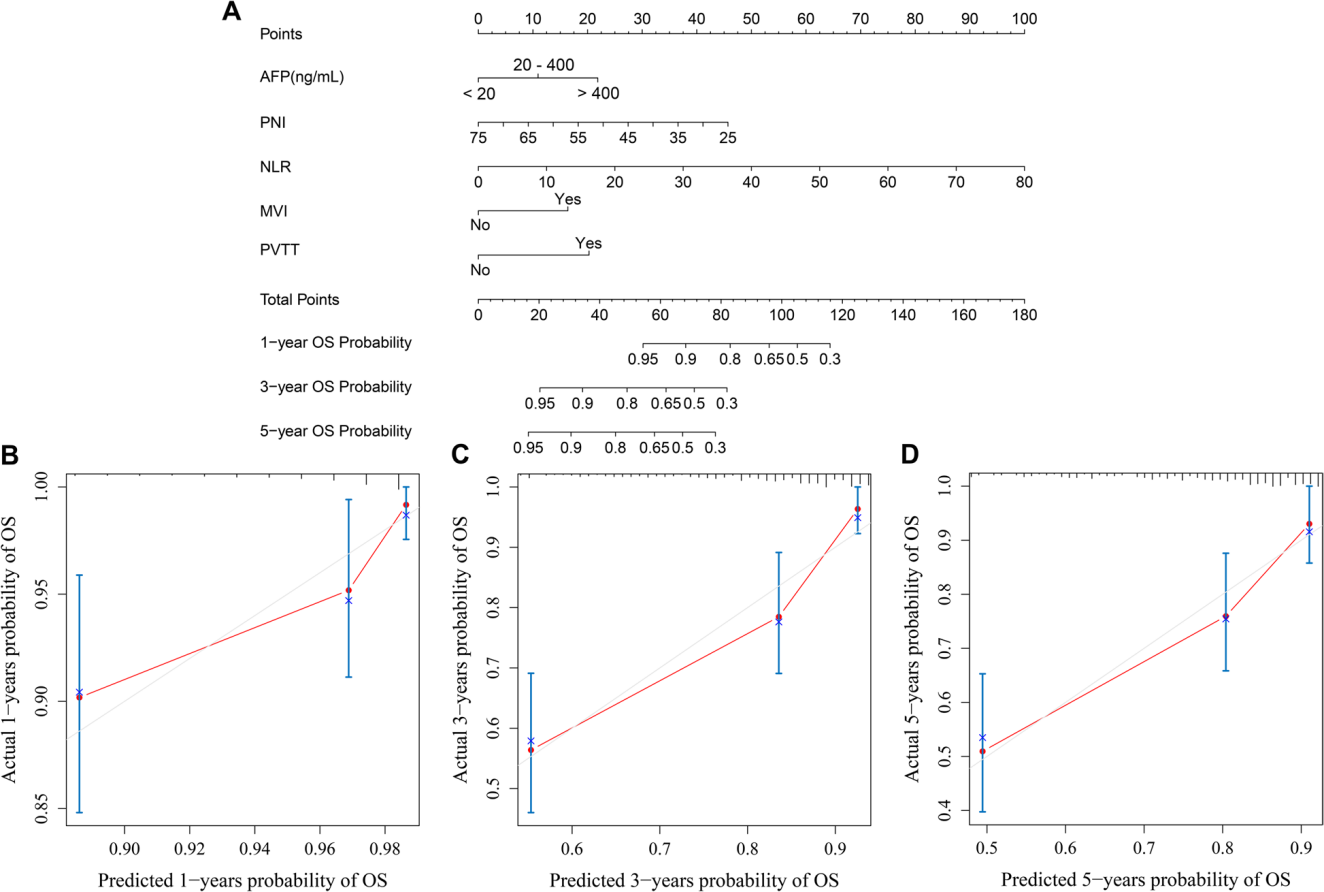
Developed prognosis nomogram model for OS. Nomogram for predicting the 1-year, 3-year and 5-year probability of OS (**A**) and Calibration curve plots of nomogram for predicting 1-year, 3-year and 5-year probability of survival (**B C D**)

We incorporated the GGT, DB, AFP, PNI, NLR, SIRI, MVI, tumour diameter and PVTT into the univariate and multivariate Cox regression equations in the OS analysis. Multivariate Cox regression analysis indicated that NLR, AFP, PNI, MVI, and PVTT were independent influencing factors for OS (Table 2). The nomogram (Fig. 3A) achieved a better concordance index of 0.772 (95% CI: 0.730-0.814) with 1000 bootstrap samples to measure discrimination in predicting 1-, 3-, and 5-year OS rates. The calibration curves for the 1-, 3- and 5-year OS rates were largely overlapped with its standard lines (Fig. 3B-D).

**Table 2:** Univariate and multivariate Cox regression analysis for OS of HCC patients baseline

Variables	Death group (*N* = 59)	Alive group (*N* = 301)	Univariate		Multivariate	
			*P*	*HR(95% CI)*	*P*	*HR(95% CI)*
GGT, U/L (≤60/>60)	23(39.0%)/36(61.0%)	181(60.1%)/120(39.9%)	0.003	2.192(1.297-3.702)	–	–
DB, umol/l (≤8/>8)	41(69.5%)/18(30.5%)	256(85.0%)/45(15.0%)	0.008	2.107(1.210-3.668)	–	–
AFP,ng/mL						
≤20	13(22.0%)	154(51.2%)		–	–	–
20-400	15(25.5%)	81(26.9%)	0.037	2.204(1.048-4.633)	0.077	1.977(0.928-4.21)
>400	31(52.5%)	66(21.9%)	<0.001	3.991(2.088-7.629)	0.001	3.093(1.589-6.023)
PNI	45.50(26.6,65.8)	49.40(26.50,73.30)	0.001	0.940(0.906-0.974)	0.014	0.953(0.917-0.99)
NLR	1.93(0.52,73.33)	2.06(0.40,25.75)	<0.001	1.072(1.037-1.107)	0.001	1.061(1.026-1.098)
SIRI	0.92(0.21,75.43)	0.85(0.22,21.45)	<0.001	1.040(1.017-1.063)	–	–
MVI(yes/no)	45(76.3%)/14(23.7%)	135(44.9%)/166(55.1%)	<0.001	3.348(1.837-6.102)	0.010	2.294(1.225-4.297)
Tumour diameter,cm	6.39±3.33	4.71±2.95	<0.001	1.129(1.064-1.198)	–	–
PVTT(yes/no)	12(20.3%)/47(79.7%)	18(6.0%)/283(94.0%)	<0.001	3.783(1.999-7.158)	0.002	2.800(1.459-5.374)

**Fig. 4:**
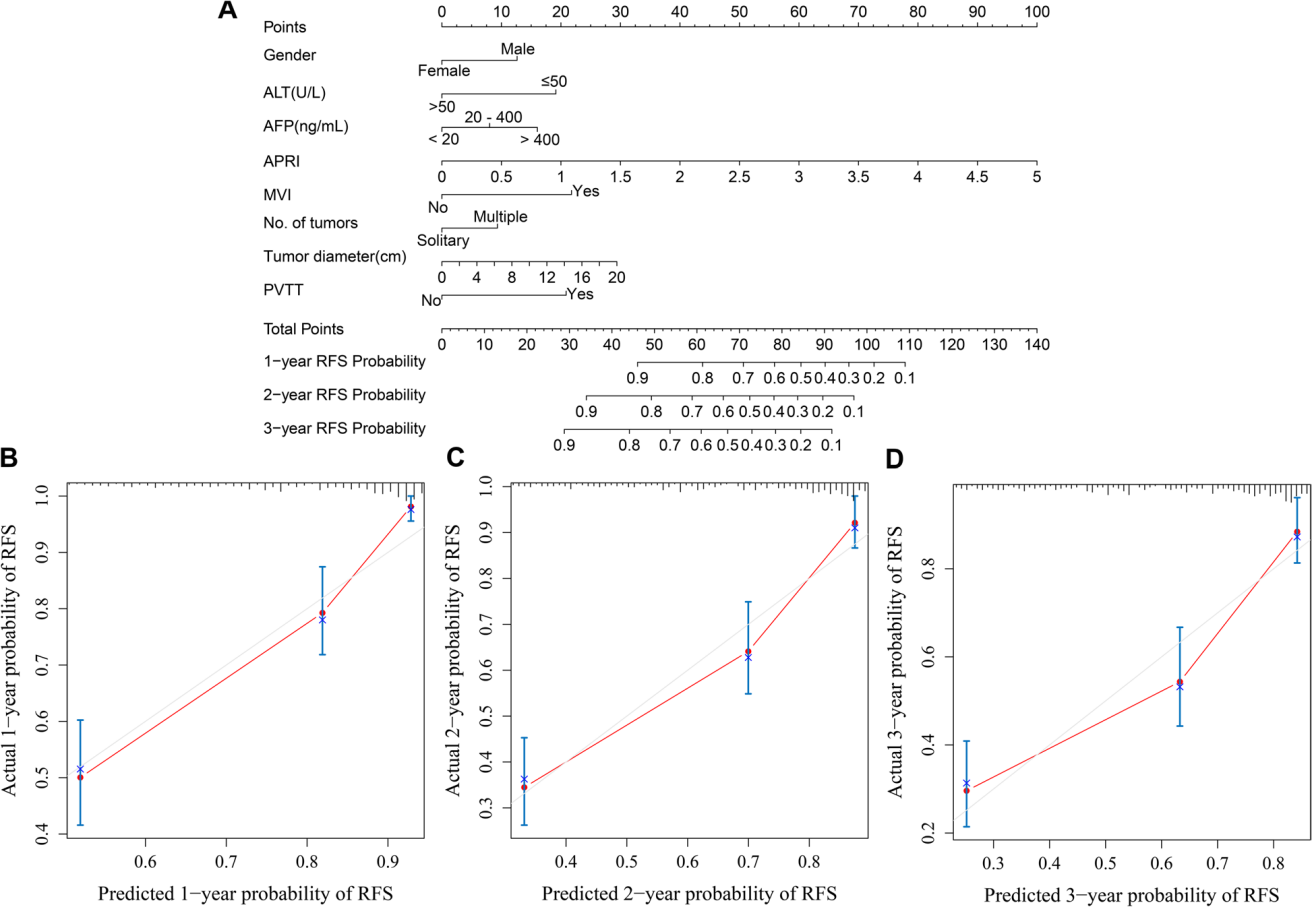
Developed prognosis nomogram model for RFS. Nomogram for predicting the 1-year, 2-year and 3-year probability of RFS (**A**) and Calibration curve plots of nomogram for predicting 1-year, 2-year and 3-year probability of RFS (**B** **C** **D**)

In the RFS analysis, gender, diabetes, ALT, ALBI, AFP, APRI, ANRI, SIRI, MVI, number of tumours, tumour diameter, tumour capsule and PVTT were incorporated into univariate and multivariate Cox regression equations. Multivariate Cox regression analysis indicated that AFP, APRI, MVI, number of tumours, tumour diameter and PVTT were independent risk factors for RFS; female and ALT were independent protective factors for RFS (Table 3). The nomogram (Fig. 4A) achieved a better concordance index of 0.774 (95% CI: 0.734-0.815) of discrimination in predicting 1-, 2-, and 3-year RFS rates. The calibration curves for the 1-, 2- and 3-year RFS rates largely overlapped with the standard lines (Fig. 4B-D).

**Table 3:** Univariate and multivariate Cox regression analysis for RFS of HCC patients baseline

Variables	Recurrence group (127)	Non-recurrence group (233)	Univariate		Multivariate	
			*P*	*HR(95% CI)*	*P*	*HR(95% CI)*
Gender(male/female)	110(86.6%)/17(13.4%)	188(80.7%)/45(19.3%)	0.130	0.674(0.404-1.123)	0.023	0.544(0.322-0.918)
Diabetes	107(84.3%)/20(15.7%)	206(88.4%)/27(11.6%)	0.099	1.497(0.927-2.416)	–	–
ALT, U/L (≤50/>50)	102(80.3%)/25(19.7%)	185(79.4%)/48(20.6%)	0.778	0.939(0.606-1.454)	<0.001	0.405(0.245-0.670)
ALBI			–	–	–	–
- 2.6	72(56.7%)	164(70.4%)	–	–	–	–
-2.60 - 1.39	53(41.7%)	67(28.8%)	0.233	0.425(0.104-1.733)	–	–
>-1.39	2(1.6%)	2(0.9%)	0.618	0.698(0.170-2.868)	–	–
AFP,ng/mL			–	–	–	–
≤20	41(32.3%)	126(54.1%)	–	–	–	–
20-400	29(22.8%)	67(28.7%)	0.174	1.391(0.864-2.239)	0.239	1.335(0.825-2.162)
>400	57(44.9%)	40(17.2%)	<0.001	2.764(1.849-4.130)	<0.001	2.161(1.402-3.290)
APRI	0.26(0.06,4.58)	0.20(0.04,2.16)	<0.001	2.062(1.428-2.976)	<0.001	2.494(1.521-4.091)
ANRI	11.87(1.51,209.06)	9.31(1.72,63.53)	<0.001	1.016(1.008-1.024)	–	–
SIRI	0.92(0.21,75.43)	0.83(0.21,51.33)	0.100	1.023(0.996-1.051)	–	–
MVI(yes/no)	95(74.8%)/32(25.2%)	85(36.5%)/148(63.5%)	<0.001	3.712(2.483-5.550)	<0.001	2.896(1.896-4.424)
Number of tumours(solitary/multiple)	93(73.2%)/34(26.8%)	197(84.5%)/36(15.5%)	0.006	1.737(1.172-2.573)	0.031	1.559(1.041-2.335)
Tumour diameter,cm	6.11±3.39	4.37±2.70	<0.001	1.139(1.091-1.190)	0.004	1.073(1.023-1.125)
Tumour capsule(yes/no)	28(22.0%)/99(78.0%)	33(14.2%)/200(85.8%)	0.047	1.534(1.005-2.339)	–	–
PVTT(yes/no)	22(17.3%)/105(82.7%)	8(3.4%)/225(96.6%)	<0.001	3.688(2.316-5.872)	<0.001	2.737(1.662-4.507)

**Fig. 5:**
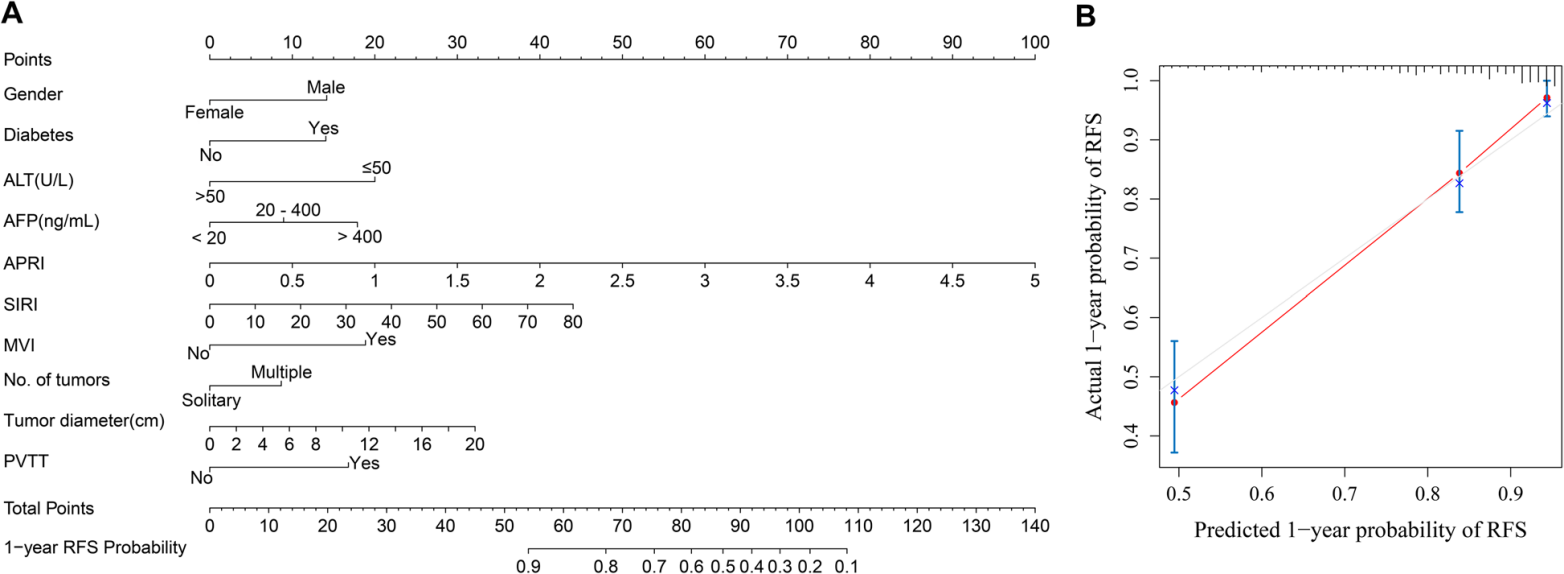
Developed prognosis nomogram model for 1-year RFS. Nomogram for predicting the 1-year probability of RFS (**A**) and Calibration curve plots of nomogram for predicting 1-year probability of RFS (**B**)

In the 1-year RFS analysis, gender, diabetes, ALT, PT, INR, AFP, PNI, APRI, ANRI, SIRI, MVI, cirrhosis, number of tumours, tumour diametes and PVTT were incorporated into the univariate and multivariate Cox regression equations. Multivariate Cox regression analysis indicated that diabetes, AFP, APRI, SIRI, MVI, number of tumours, tumour diameter and PVTT were independent risk factors for 1-year RFS; female and ALT were independent protective factors for 1-year RFS (Table 4). Incorporating diabetes, AFP, APRI, SIRI, MVI, number. of tumours, tumour diameter, PVTT, gender, and ALT, the nomogram (Fig. 5A) achieved a better concordance index of 0.809 (95% CI: 0.766-0.852) in regards to discrimination in predicting 1-year RFS rates. The calibration curves for the 1-year RFS rates largely overlapped with the standard lines (Fig. 5B).

**Table 4:** Univariate and multivariate Cox regression analysis for 1-year RFS of HCC patients baseline

Variables	1-year recurrence group (81)	Non-recurrence group (279)	Univariate		Multivariate	
			*P*	*HR(95% CI)*	*P*	*HR(95% CI)*
Gender(male/female)	71(87.7%)/10(12.3%)	227(81.4%)/52(18.6%)	0.170	0.629(0.325-1.220)	0.025	0.455(0.228-0.906)
Diabetes(yes/no)	17(21.0%)/64(79.0%)	30(10.8%)/249(89.2%)	0.016	1.930(1.130-3.297)	0.005	2.210(1.264-3.866)
ALT, U/L (≤50/>50)	66(81.5%)/15(18.5%)	221(79.2%)/58(20.8%)	0.569	0.850(0.485-1.489)	0.001	0.334(0.175-0.635)
PT, seconds (≤13/>13)	70(86.4%)/11(13.6%)	233(83.5%)/46(16.5%)	0.633	0.856(0.453-1.617)	–	–
INR(≤1/>1)	20(24.7%)/61(75.3%)	65(23.3%)/214(76.7%)	0.868	0.958(0.578-1.588)	–	–
AFP, ng/mL			–	–	–	–
≤20	21(25.9%)	146(52.3%)	–	–	–	–
20-400	21(25.9%)	75(26.9%)	0.044	1.860(1.016-3.406)	0.040	1.923(1.032-3.583)
>400	39(48.2%)	59(20.8%)	<0.001	3.520(2.07-5.983)	<0.001	2.805(1.602-4.911)
PNI	46.4(26.6,65.8)	49.5(26.5,73.3)	0.004	0.953(0.923-0.985)	–	–
APRI	0.30(0.07,4.58)	0.21(0.04,2.16)	<0.001	2.072(1.421-3.020)	<0.001	2.86(1.764-4.637)
ANRI	13.33(1.51,209.06)	9.8(1.72,63.53)	<0.001	1.016(1.007-1.025)	–	–
SIRI	0.92(0.21,75.43)	0.83(0.21,51.33)	0.039	1.027(1.001-1.052)	0.021	1.032(1.005-1.060)
MVI(yes/no)	62(76.5%)/19(13.5%)	118(42.3%)/161(57.7%)	<0.001	3.572(2.136-5.974)	<0.001	2.889(1.662-5.021)
Cirrhosis(yes/no)	42(5.9%)/39(48.1%)	131(47.0%)/148(53.0%)	0.265	1.282(0.829-1.983)	–	–
Number of tumours(solitary/multiple)	25(30.9%)/56(69.1%)	45(16.1%)/234(83.9%)	0.003	2.052(1.280-3.289)	0.054	1.621(0.991-2.652)
Tumour diameter,cm	6.49±3.53	4.55±2.78	<0.001	1.154(1.094-1.217)	0.002	1.098(1.034-1.165)
PVTT(yes/no)	19(23.5%)/62(76.5%)	11(3.9%)/268(96.1%)	<0.001	4.189(2.504-7.008)	0.001	2.503(1.431-4.378)

**Fig. 6:**
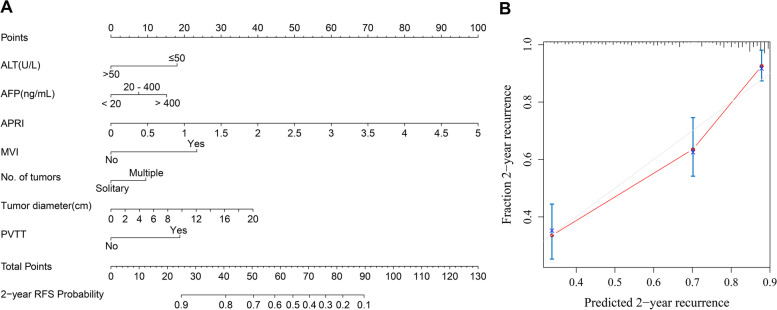
Developed prognosis nomogram model for 2-year RFS. Nomogram for predicting the 2-year probability of RFS (**A**) and Calibration curve plots of nomogram for predicting 2-year probability of RFS (**B**)

In the 2-year RFS analysis, ALT, ALBI, AFP, PNI, APRI, MVI, number of tumours, tumour diameter, tumour capsule and PVTT were incorporated into the univariate and multivariate Cox regression equations. Multivariate Cox regression analysis indicated that AFP, APRI, MVI, number of tumours, tumour diameter and PVTT were independent risk factors for 2-year RFS; ALT was an independent protective factor of 2-year RFS (Table 5). The nomogram (Fig. 6A) achieved a better concordance index of 0.756 (95%CI: 0.696-0.816) with 1000 bootstrap samples to measure discrimination in predicting 2-year RFS rates. The calibration curves for the 2-year RFS rates largely overlapped (Fig. 6B).

**Table 5:** Univariate and multivariate Cox regression analysis for 2-year RFS of HCC patients baseline

Variables	2-year recurrence group (*N *= 112)	Non-recurrence group (*N *= 248)	Univariate		Multivariate	
			*P*	*HR(95% CI)*	*P*	*HR(95% CI)*
ALT, U/L (≤50/>50)	89(79.5%)/23(20.5%)	198(79.8%)/50(20.2%)	0.883	0.966(0.611-1.528)	0.002	0.437(0.259-0.740)
ALBI				–	–	–
-2.6	59(52.7%)	177(71.4%)		–	–	–
-2.60 - 1.39	51(45.5%)	69(27.8%)	<0.001	1.952(1.342-2.841)	–	–
>-1.39	2(1.8%)	2(0.8%)	0.127	3.001(0.733-12.292)	–	–
AFP,ng/mL						
≤20	34(30.4%)	133(43.6%)		–	–	–
20-400	26(23.2%)	70(28.2%)	0.130	1.484(0.891-2.474)	0.240	1.361(0.814-2.275)
>400	52(46.4%)	45(18.1%)	<0.001	2.987(1.938-4.604)	0.002	2.040(1.305-3.191)
PNI	46.7(26.6,65.8)	49.7(26.5,73.3)	0.001	0.952(0.926-0.979)	–	–
APRI	0.27(0.07,4.58)	0.20(0.04,2.16)	<0.001	2.006(1.421-2.986)	0.001	2.447(1.460-4.102)
MVI(yes/no)	86(76.8%)/26(23.2%)	94(37.9%)/154(62.1%)	<0.001	3.905(2.517-6.060)	<0.001	3.027(1.909-4.799)
Number of tumors(solitary/multiple)	80(71.4%)/32(28.6%)	210(84.7%)/38(15.3%)	0.003	1.858(1.233-2.801)	0.039	1.555(1.022-2.365)
Tumor diameter,cm	6.30±3.45	4.39±2.69	<0.001	1.148(1.097-1.202)	0.001	1.095(1.004-1.152)
Tumor capsule(yes/no)	27(24.1%)/85(75.9%)	34(13.7%)/214(86.3%)	0.041	1.570(1.018-2.421)	–	–
PVTT(yes/no)	21(18.8%)/91(81.3%)	9(3.6%)/239(96.4%)	<0.001	3.666(2.274-5.91)	0.001	2.402(1.453-3.970)

The resultant nomograms could accurately preoperatively distinguish the death and recurrence of HCC patients and had better consistency between the predicted probability and the observed frequency of OS, RFS, 1-year RFS, and 2-year-RFS rates.

### Risk stratification for survival and recurrence based on the NomoScores and prognostic assessment

**Fig. 7:**
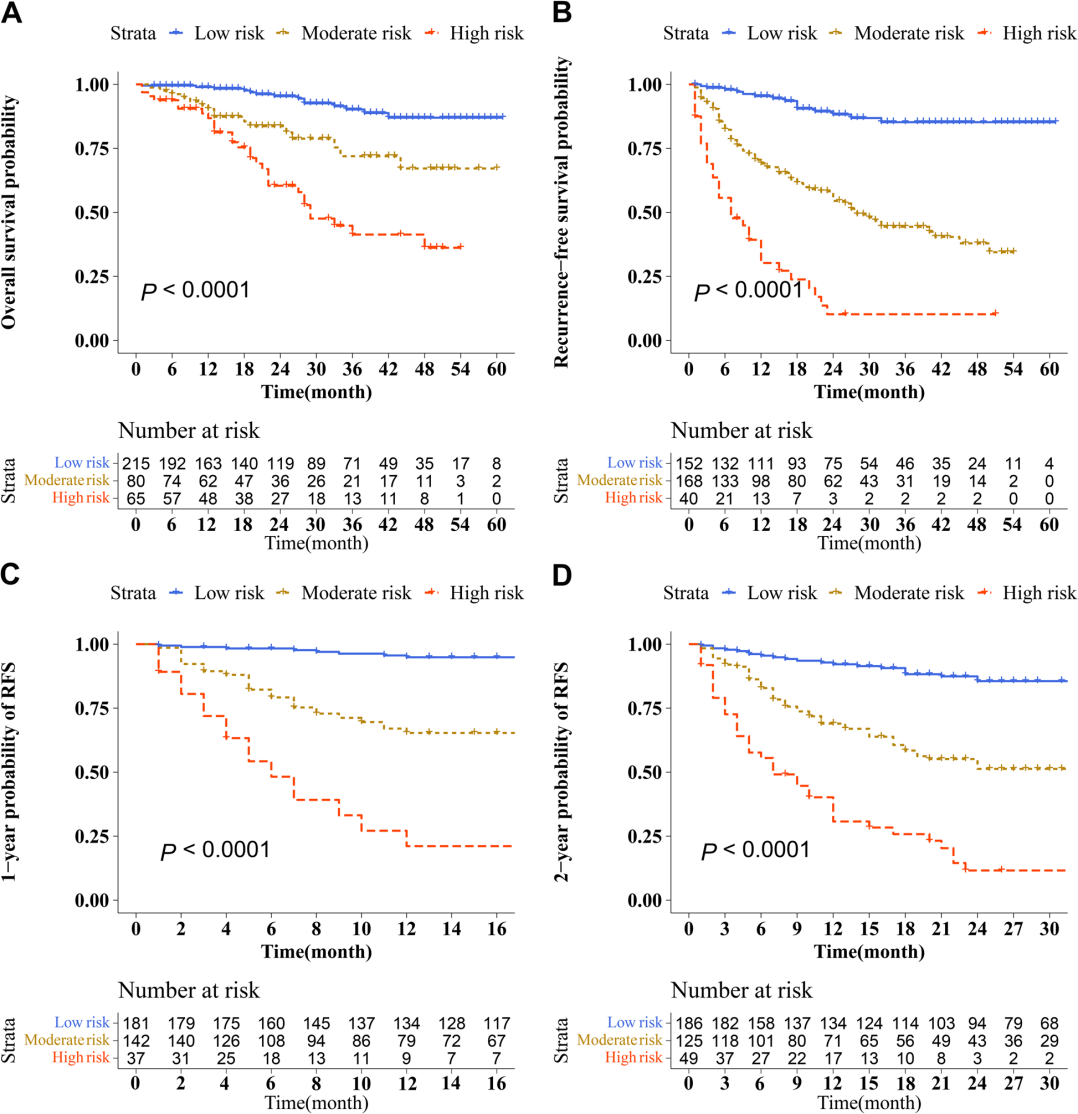
Risk stratification for survival and recurrence based on the nomogram risk scores. Risk stratification for OS (**A**), and Risk stratification for RFS (**B**), and Risk stratification for 1-year RFS (**C**), and Risk stratification for 2-year RFS (**D**)

Hepatectomy patients were divided into different risk groups based on the total risk NomoScores calculated by the nomogram models for judging the discriminatory abilities of the nomograms for OS, overall recurrence, 1-year recurrence, and 2-year recurrence. The optimal cut-off points were auto-calculated by X-tile software. In OS groups(high-risk group: > 64; moderate-risk group: 49-64; low-risk group: < 49), RFS groups(high-risk group: > 85; moderate-risk group: 52-85; low-risk group: < 52), 1-year RFS groups(high-risk group: > 90; moderate-risk group: 62-90; low-risk group: < 62), and 2-year RFS groups(high-risk group: > 74; moderate-risk group: 59-74; low-risk group: < 59), the risk NomoScores calculated can divide HCC patients into the high-, moderate- and low-risk groups. Survival curves were calculated to compare OS and RFS, 1-year RFS, and 2-year RFS rates in three different risk groups were determined using the Kaplan–Meier method, and the results showed a significant discriminatory ability for death, overall recurrence, 1-year recurrence, and 2-year recurrence risks in the patients based on the NomoScores (*P* < 0.05, Fig. 7).

**Fig. 8:**
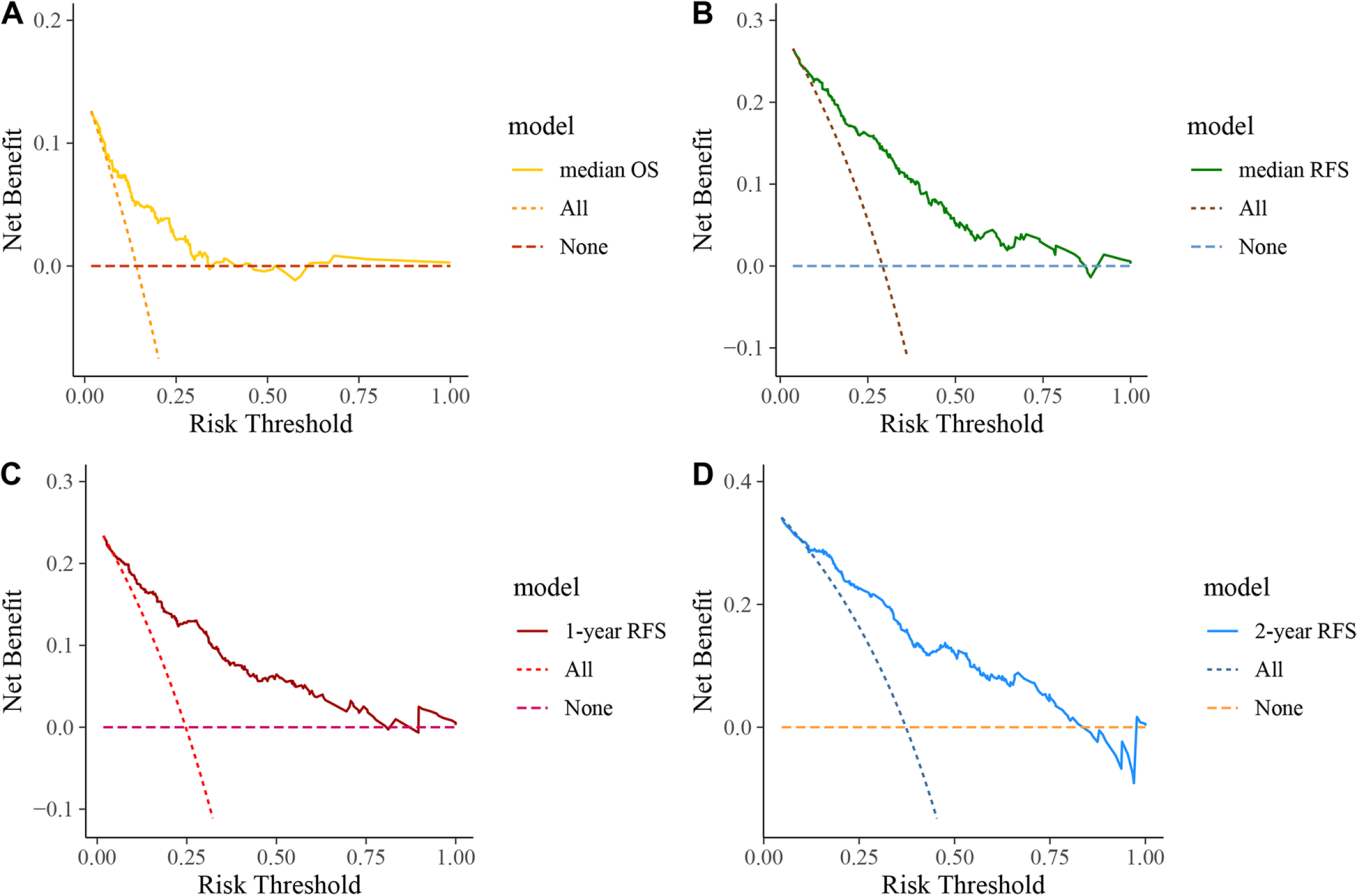
The decision curve analysis for developed nomogram model. DCA for OS (**A**), and DCA for RFS (**B**), and DCA for 1-year RFS (**C**), and DCA for 2-year RFS (**D**)

DCA is usually used to evaluate the clinical net benefit of the prediction models. In this study, DCA revealed that the nomogram models could augment net benefits and exhibited a wider range of threshold probabilities in predicting OS, RFS, 1-year RFS, and 2-year-RFS rates (Fig. 8). The median OS and RFS times were selected as the cut-off values in Fig. 8A and B.

## Discussion

In our study, nine inflammatory markers that can be easily calculated preoperatively via complete blood count and liver function tests were filtered to construct nomogram prediction models for the prognosis of HCC patients after curative resection. Finally, the results showed that PNI and NLR were independent risk factors for OS, and in the recurrence groups, APRI was a common predictive factor among RFS, 1-year RFS, and 2-year RFS. SIRI was an independent prognostic factor for 1-year RFS in HCC patients after curative resection. There was an apparent difference in the consequential inflammatory factors between OS and recurrence. Overall, lower cost, simplicity of the test, and fewer traumatic inflammatory markers showed excellent predictive ability when combined with AFP, tumour diameter, MVI, PVTT, etc.

The liver is a central immunomodulator that ensures organ and systemic protection while maintaining immunotolerance [25]. Deregulation of the balanced liver immunology network is a hallmark of chronic liver disease and HCC. As a prototypical inflammation-related cancer, approximately 90% of HCC burden is associated with prolonged hepatitis due to viral hepatitis, excessive alcohol intake, nonalcoholic fatty liver disease (NAFLD) or nonalcoholic steatohepatitis (NASH) [26]. The immune microenvironment and inflammatory markers are all parts of the systemic inflammatory response and play an important role in cancer development, progression, invasion, and metastasis [10, 27].

In early studies, PNI was utilized to evaluate the immunological and nutritional aspects of carcinoma patients, predominantly as an indicator to investigate the nutritional status of patients [20, 28]. Daly et al. [29] reported that higher rates of postoperative complications and mortality were associated with lower serum albumin levels and lymphocyte count in the peripheral blood of carcinoma patients. Lymphocytes can guard against tumorigenesis and recurrence and regulate immune function by expressing cytokines, thus inducing the effect of cytotoxic death, and altering the tumour microenvironment [30]. Pinato et al. [31] reported that the presence of a systemic inflammatory response, as measured by the PNI, was an independent predictor of poor OS in HCC patients. The NLR has been proven to be a significant prognostic factor of HCC in several types of cancers [32–35]. Mano Y et al. [36] revealed that a higher preoperative NLR level was correlated with the accumulation of tumour-associated macrophages, and tumour-associated macrophages were related to tumour aggressiveness in HCC patients [37]. The proinflammatory cytokines interleukin-6, expressed by tumour-associated macrophages, have been reported to be important to the development and progression of HCC by activating the transcription factor STAT3 and NF-κB pathways [38, 39]. Our study demonstrated that PNI and NLR were independent prognostic factors to predict postoperative OS in HCC patients,consistent with other studies [31, 40–42]. Although previous studies reported that PNI and NLR were important prognostic factors of HCC recurrence [43, 44], this was not found in our study.

In addition, we found that APRI was the collective prediction factor for RFS, 1-year RFS, and 2-year RFS. In chronic hepatitis B and C infection, studies demonstrated that APRI could be regarded as a strong indicator of liver diseases with significant and advanced fibrosis and cirrhosis [45, 46]. Furthermore, as reported by Shen SL et al. [47], APRI was associated with adverse characteristic features and poor prognosis in HCC, especially for patients with HBV infection or cirrhosis. Liu Y et al. [48] demonstrated that APRI was a significant independent adverse prognostic factor predicting early and late recurrence of hepatocellular carcinoma after curative hepatectomy. APRI levels may reflect both inflammatory liver environment that promotes tumour invasion and continuous liver damage which predisposes patients to de novo tumours, and is a factor significant to tumour recurrence. In our study, 86% of HCC patients were HBV positive. Furthermore, to the best of our knowledge, our study is the first to identify SIRI as an independent prediction factor of 1-year RFS in HCC hepatectomy patients. In a previous study, a high SIRI value was associated with poor overall survival, but the relationship between SIRI and RFS was not explored [16]. In postoperative adjuvant chemotherapy, high SIRI indicated poor OS and RFS as an independent predictive factor in pancreatic ductal adenocarcinoma and breast cancer [49, 50]. Systemic chronic inflammation plays a significant role in the development and progression of HCC. As such, systemic immune competence may influence patient prognosis through the local immune response. However, the mechanisms through which inflammatory markers can predict the prognosis of HCC patients are complicated and not very clear at present. Future studies are needed to elucidate the underlying mechanisms.

Feature selection was an important step to eliminate overfitting when faced with so many features. Overfitting, optimism, and miscalibration might be addressed and accounted for during the model development by applying bootstrapping techniques and LASSO regression [51]. The nomograms constructed in our study achieved better concordance indexes of 0.772, 0.774, 0.809, and 0.756 in predicting OS, RFS, 1-year RFS, and 2-year RFS respectively. The resultant nomograms can significantly identify patients with different prognoses and are suitable for various patients based on risk scores. A majority of previous studies generally randomly split the dataset into two subsets, a development dataset and a validation dataset, which was not done in this study. This approach was statistically inefficient and/or wasteful as not all available data were utilized in the development of the model [52]. Internal validation was a necessary part of prediction model development [53]. However, for external validation, substantial sample sizes should be used for sufficient power to detect clinically important changes in performance as compared with the internally validated estimate [54]. Consequently, limited to the sample size, we were constrained to internal validation. However, external validation was more persuasive than internal validation in verifying the stability of the nomogram model. DCA was also performed and revealed that the nomograms have a good clinical utility.

Notable limitations in our study include the following. First, the study was a single-centre retrospective cohort, and a longer follow-up time is required. Second, we performed internal validation solely, and external validations of multicentre studies are still needed and indispensable. Last but not least, the heterogeneity in the therapy of HCC patients after hepatectomy may lead to different clinical prognoses.

## Conclusions

In conclusion, the constructed nomograms showed high predictive accuracy for OS, RFS, 1-year RFS, and 2-year RFS in HCC patients after surgical resection. The nomograms could be useful clinical tools to guide a rational and personalized treatment approach and prognosis judging.

## Data Availability

The datasets used or analyzed during the current study are available from the corresponding author on reasonable request. In order to protect study participants’ privacy, our data cannot be shared openly.
